# Supplementary material for: ISG15‐Dependent Stabilisation of USP18 Is Necessary but Not Sufficient to Regulate Type I Interferon Signalling in Humans
Source: Eur J Immunol. 2025 Feb 11;55(2):e202451651. doi: 10.1002/eji.202451651 (PMC11811815; doi:10.1002/eji.202451651)
Supplement: Supplementary file 1 — Supporting Information [file EJI-55-e202451651-s002.pdf]

Supplementary Material

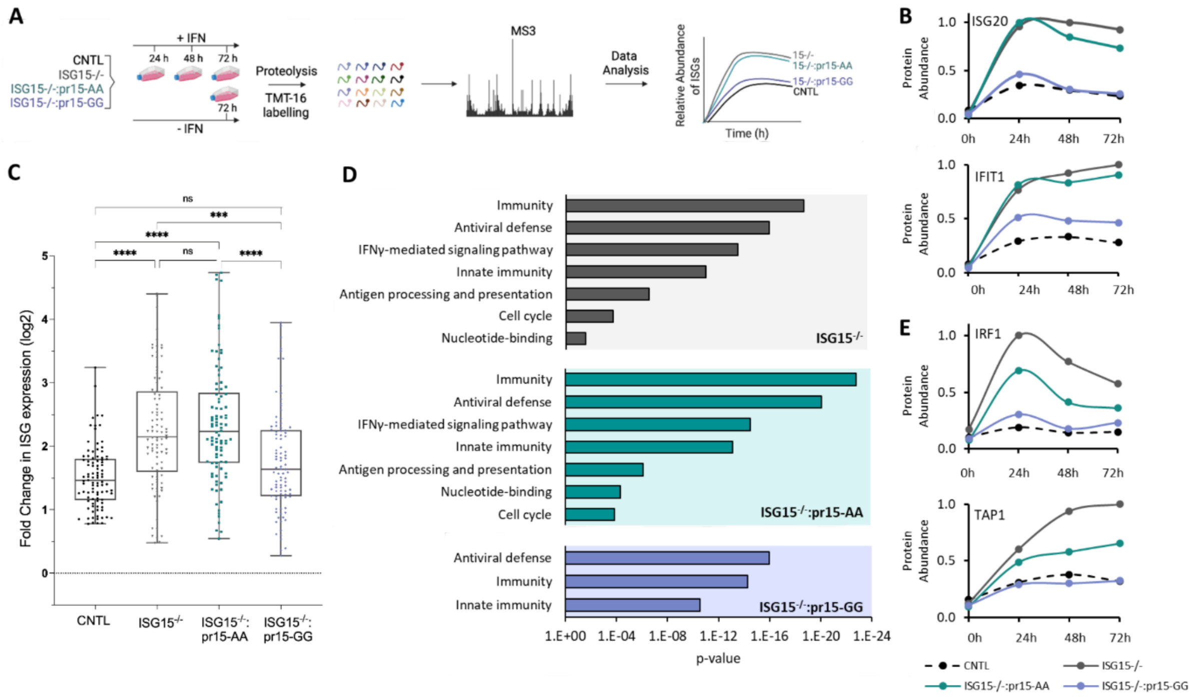

Supplementary figure 1. Quantitative temporal analysis of proteomes from IFN-α-treated ISG15$^{-/-}$ and reconstituted cells. (A) Schematic presentation of the experimental workflow. **(B)** Abundance of representative examples of well-known ISGs over time after IFN-α simulation, normalised to a maximum of 1 for each protein. **(C)** Comparative quantitative analysis of ISG expression in A549 (CNTL), A549-ISG15$^{-/-}$ and ISG15.AA- or ISG15.GG-expressing cells. ISGs were defined by (a) correspondence with the Interferome database and (b) >1.7-fold increase in abundance in IFN-a stimulated compared to unstimulated A549 control cells. Statistical significance was assessed using one-way ANOVA and Tukey multiple comparisons test; ***, $p < 0.001$, ****, $p < 0.0001$, n.s., no statistical significance**. (D)** Pathway analysis using DAVID software. Proteins >1.7-fold enriched following IFN-α treatment in each cell type compared to A549 control cells were searched using DAVID software using the default medium classification stringency. Benjamini-Hochberg adjusted p-values are shown. **(E)** Expression profiles of representative examples of well-characterised factors with roles in IFN-γ signalling and antigen processing and presentation pathways. Protein abundance was calculated as described in (B).

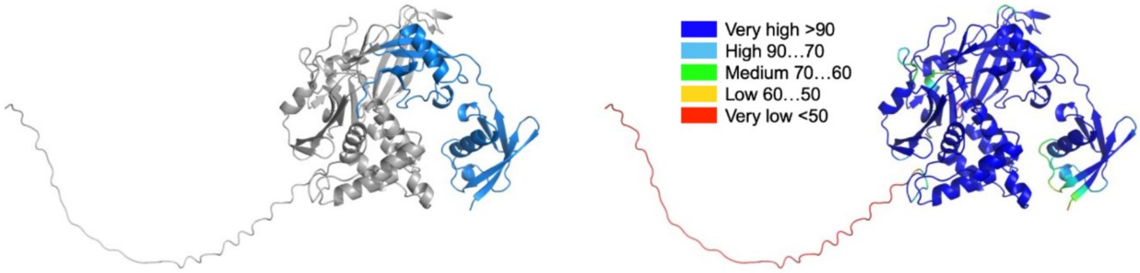

Supplementary figure 2. AlphaFold2 predicts human USP18:ISG15 complex wit high confidence. AlphaFold2 prediction of human USP18 in complex with ISG15, obtained via ColabFold [30]. The top-ranked structure is shown, coloured by chain (left, grey, USP18, blue, ISG15) and coloured by the per-residue confidence metric pLDDT (right).

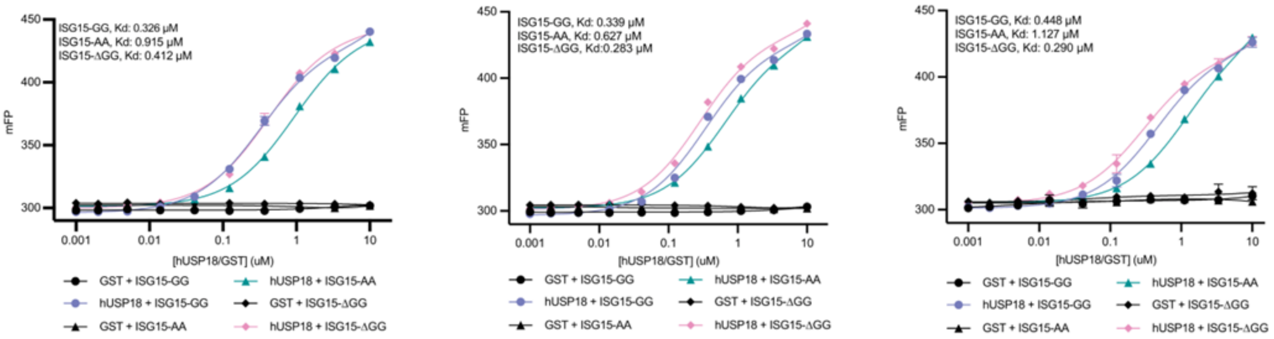

Supplementary figure 3. Binding assays demonstrate C-termini of ISG15 contribute to high affinity interactions. Three independent fluorescence polarisation (FP) binding assays of human USP18 to ISG15 and ISG15 variants. GST was used as a negative control.

Supplementary File 1. Interactive spreadsheet of quantitative proteomics data. The 'Data' worksheet presents minimally annotated protein data. The 'Plotter' worksheet allows relative protein abundance of any quantified protein to be easily visualised. Input gene name (up to 2 entries) to plot relative protein abundance at each time point. The protein with the highest abundance is set to 1.
